# Pulmonary Embolism after Operations upon the Bladder and Prostate

**Published:** 1902-10

**Authors:** 


### Pulmonary Embolism After Operations Upon the Bladder and Prostate.

Edward L. Keyes, Jr., in *New York Medical Journal,* gives ten cases of sudden death, presumably from pulmonary embolism. One of these cases followed a suprapubic lithotomy, one a suprapubic cystotomy and the other eight various operations on the prostate. Bearing in mind the sluggish circulation in the pelvic veins, and the lowered vitality met in most of these patients, the question is asked if danger of embolism after these operations be not more than a chimera, and an appeal is made for a careful study of the conditions and results attending these operations.

